# New Target Genes for the Peroxisome Proliferator-Activated Receptor-*γ* (PPAR*γ*) Antitumour Activity: Perspectives from the Insulin Receptor

**DOI:** 10.1155/2009/571365

**Published:** 2009-06-29

**Authors:** Daniela P. Foti, Francesco Paonessa, Eusebio Chiefari, Antonio Brunetti

**Affiliations:** ^1^Department of Clinical and Experimental Medicine “G. Salvatore”, University of Catanzaro “Magna Græcia”, V.le Europa (Loc. Germaneto), 88100 Catanzaro, Italy; ^2^Chair of Clinical Pathology, University of Catanzaro “Magna Græcia”, V.le Europa (Loc. Germaneto), 88100 Catanzaro, Italy; ^3^Chair of Endocrinology, University of Catanzaro “Magna Græcia”, V.le Europa (Loc. Germaneto), 88100 Catanzaro, Italy

## Abstract

The insulin receptor (IR) plays a crucial role in mediating the metabolic and proliferative functions triggered by the peptide hormone insulin. There is considerable evidence that abnormalities in both IR expression and function may account for malignant transformation and tumour progression in some human neoplasias, including breast cancer. PPAR*γ* is a ligand-activated, nuclear hormone receptor implicated in many pleiotropic biological functions related to cell survival and proliferation. In the last decade, PPAR*γ* agonists—besides their known action and clinical use as insulin sensitizers—have proved to display a wide range of antineoplastic effects in cells and tissues expressing PPAR*γ*, leading to intensive preclinical research in oncology. PPAR*γ* and activators affect tumours by different mechanisms, involving cell proliferation and differentiation, apoptosis, antiinflammatory, and antiangiogenic effects. We recently provided evidence that PPAR*γ* and agonists inhibit IR by non canonical, DNA-independent mechanisms affecting IR gene transcription. We conclude that IR may be considered a new PPAR*γ* “target” gene, supporting a potential use of PPAR*γ* agonists as antiproliferative agents in selected neoplastic tissues that overexpress the IR.

## 1. Structure and Biological Function of the IR

**Figure 1:**
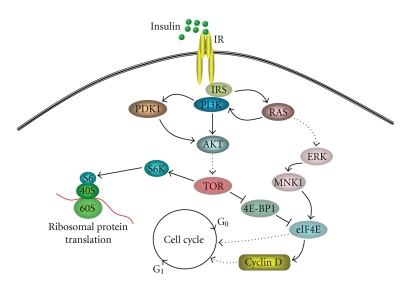
Upon binding of insulin, the IR undergoes autophosphorylation which enables the receptor to have a kinase activity and phosphorylates various cytoplasmic substrates, such as IRSs. From this point, signaling proceeds via a variety of signaling pathways (i.e., PI3K signaling pathway, Ras and MAP kinase cascade) that are responsible for the metabolic, growth-promoting and mitogenic effects of insulin.

The peptide hormone insulin regulates the metabolism and growth of most cells [1]. In target tissues, it is involved in anabolic processes to produce proteins, polysaccharides, nucleic acids, and lipids. For this complex task, its action implicates three major sites of metabolic regulation. At the plasma membrane, insulin increases the transport of ions, glucose, and other substrates; in the cytoplasm and its organelles, it activates a numer of intracellular enzymes, such as glycogen synthase; in the nucleus, insulin regulates the synthesis of RNA and DNA. The first step in insulin action is its binding to the IR, a phylogenetically ancient receptor tyrosine kinase protein embedded in the plasma membrane of virtually all cells [2–5]. Therefore, the IR plays a critical role in both directing the hormone to a specific target tissue and programming the biological response of the tissue to the hormone. The IR belongs to the tyrosine kinase growth factor receptor family. When insulin binds to the IR, the receptor becomes activated and induces a cascade of intracellular events that will lead to several metabolic and growth promoting effects. The IR consists of two identical extracellular alpha subunits (130 kDa) that house insulin binding domains, and two transmembrane beta subunits (95 kDa) that contain ligand activated tyrosine kinase activity in their intracellular domains [2–5]. A further understanding of the nature of the IR and its relationship to other receptors has been provided by the cloning of the human IR gene [6–8]. Upon binding of insulin to the alpha subunits, the receptor is first activated by tyrosine autophosphorylation, and then the IR tyrosine kinase phosphorylates various intracellular effector molecules (such as IRSs) which in turn alters their activity, thereby generating a biological response [3–5]. In this context, a big effort has been made by scientists to unravel intracellular signaling pathways involving metabolic or mitogenic responses [9, 10]. A bulk of evidences demonstrate that mitogenic stimuli triggered by growth factors are able to regulate different cell-cycle checkpoints [11]. Insulin stimulation activates the IR/IRS/PI3K/PDK1 pathway, leading to the activation of S6K, which is crucial for ribosome biosynthesis, and necessary for G_0_-G_1_ transition. S6k is also stimulated by TOR, which induces the translation of cell-cycle regulators, such as cyclin D, mediating progression through the G_1_ phase. Cyclin D is also a target for the Ras/ERK cascade induced by insulin, leading to a synergistic effects on cell proliferation [10–12] (Figure 1).

The interaction between IR and other ligands of the IGF (insulin-like growth factor) system [13] implicates an even more complex scenario. The IR exists as two splice variant isoforms: the IR-B isoform that is responsible for signaling metabolic responses involved mainly in the regulation of glucose uptake and metabolism by increasing glucose transporter molecules on the plasma membrane of the insulin-responsive tissues muscle, liver, and fat, and the IR-A isoform, that is expressed in certain tumours (such as mammary cancers), signals predominantly mitogenic responses and is capable of binding IGF-II with high affinity [14, 15]. As a consequence of these cellular activities, abnormalities of IR expression and/or function can facilitate the development of several metabolic and neoplastic disorders in humans as well as in animal models. In addition, hybrid heterodimeric receptors consisting of insulin and IGF-I receptor subunits may form and could play a role in receptor signaling in normal and abnormal tissues [13].

## 2. Molecular and Clinical Significance of the IR in Cancer

Dysfunctional IR signaling is implicated in certain common dysmetabolic disorders, including obesity, type 2 diabetes, the dysmetabolic syndrome X, and the polycystic ovary syndrome [16–19]. Also, clinical syndromes due to mutations in the IR gene have been identified in patients with genetic forms of severe insulin resistance [20, 21] A relation between IR and cancer has been established following the observation that overexpression of functional IRs can occur in human breast cancer and other epithelial tumours including ovarian and colon cancer, in which the IR may exert its oncogenic potential via abnormal stimulation of multiple cellular signaling cascades, enhancing growth factor-dependent proliferation and/or by directly affecting cell metabolism [22–27]. On the other hand, epidemiological and clinical evidence points to a link between insulin resistant syndromes such as obesity and type 2 diabetes and cancer of the colon, liver, pancreas, breast and endometrium. The mechanistic link between insulin resistance and cancer is unknown, but constitutive activation of the tyrosine kinase activity of IR and related downstream signaling pathways by chronic sustained hyperinsulinemia (a hallmark of insulin resistance) in these clinical syndromes appears to have a role in the neoplastic transformation process [28–30]. Mechanisms due to hyperinsulinemia that promote malignancy and neoplastic progression include the increase in IGF-I and sex hormones bioavailability, the increase in proinflammatory cytokines, and oxidative stress. Although the molecular mechanisms that cause neoplastic transformation, sustaining tumor progression in the presence of IR hyperexpression and/or hyperstimulation are not fully understood, an explanation for increased IR expression in epithelial tumours has been recently provided by us for several human breast cancers, in which overexpression of the transcription factor AP2-*α* (activator protein 2-*α*) accounted for increased IR expression in neoplastic breast tissue [31]. In these cases, it has been demonstrated that transactivation of the IR gene by AP-2*α* represented a fundamental prerequisite for AP-2*α* to activate IR gene transcription in neoplastic breast tissue. Similarly, a functional link between IR, and cyclin D1 has been recently described in pancreatic cancer [32]. Thus, a better understanding of the mechanisms responsible for increased IR expression may contribute to identify new therapeutic targets for tumours with abnormal IR expression and/or function [22, 33].

## 3. Regulation of the IR Gene Expression

In target cells the IR has been shown to be under the regulation of hormones, metabolites, and differentiation [34]. To further understand the regulation of the IR, we and others have identified and analyzed the IR promoter region [6–8]. This region extends over 1,800 bases 5’ upstream from the IR gene ATG codon. The IR promoter has no TATA or CAAT boxes, and includes multiple transcription initiation sites, reflecting a feature common to the promoters of many constitutively expressed genes (so-called housekeeping genes). The IR is expressed at low levels in most cells and this expression appears to be driven in part by a series of GGGCGG repeats (in the region −400 to −650), which are putative binding sites for the mammalian transcription factor Sp1. The IR is expressed at higher levels in differentiated target tissues such as muscle and fat. At these levels, tissue-specific and ubiquitous nuclear transcription factors cooperate to induce IR gene transcription. We have previoulsy identified two distinct, functionally active DNA sequences, C2 and E3, within the IR gene promoter, which had a significant ability to drive IR gene transcription [8]. The molecular mechanisms regulating IR gene expression are being elucidated and evidence has been provided showing that the architectural transcription factor (High Mobility Group A1 Protein) HMGA1 is required for proper transcription of the IR gene in cells expressing IRs. HMGA1 acts on the IR gene promoter as an element necessary for the formation of a transcriptionally active multiprotein–DNA complex involving, in addition to the HMGA1 protein, the ubiquitously expressed transcription factor (specificity protein 1) Sp1, and (CCAAT-enhancer binding protein beta) C/EBP*β*. By potentiating the recruitment and binding of Sp1 and C/EBP*β* to the IR promoter, HMGA1 greatly enhances the transcriptional activities of these factors in the context of the IR gene [35, 36]. Transcriptional activation of the human IR gene by HMGA1, Sp1, and C/EBP*β* requires the assembly and cooperation among these various nuclear factors at the levels of the two AT-rich sequences of the IR gene promoter, C2 and E3, which have a significant ability to drive transcription when introduced into mammalian cells [8, 35, 36]. Qualitative or quantitative defects in these binding proteins and/or abnormalities in their consensus sequences within the IR gene may affect IR transcription, leading to abnormalities in IR gene and protein expression [21]. Overexpression of IR in cells which normally express low levels of IR, like epithelial cells, may increase biological responses to insulin and trigger a ligand-mediated neoplastic transformation.

## 4. Pleiotropic Effects of PPAR*γ*

PPAR*γ* is a nuclear hormone receptor [37–39] highly expressed in adipose tissue, where it regulates adipogenesis and the multiple functions of mature adipocytes [40–42]. It is also expressed in many other tissues and cell types, including liver, skeletal muscle, breast, prostate, colon, lung, monocytes, macrophages, and B lineage cells [43–46]. Besides a fundamental role in lipid metabolism, PPAR*γ* plays a number of pleiotropic effects, that include a critical role in placental vascularization, myocardial health, and embryonic development [47, 48]. It also influences the production of cytokines, growth factor release, cell cycle progression, leading to differentiation-inducing, anti-inflammatory and anti-proliferative effects [49–51]. PPAR*γ* generally exterts its biological function as heterodimer with the retinoid-X-receptor through binding to a specific cis-acting sequence on DNA—a peroxisome proliferator response element (PPRE)—to initiate transcription [37, 50]. To act as transcription factor, PPAR*γ* requires activation by binding its ligand. The PPAR*γ* activators identified so far include naturally occurring compounds, as some unsaturated fatty acids, some anti-inflammatory molecules—such as a prostaglandin J2 metabolite (15-deoxy-D12,14-PGJ2)—, oxidized low-density lipoprotein (LDL) particles 9-and 13-HODE (hydroxyoctadeca-10E,12Z-dienoic acid) [37, 49], and synthetic compounds, mainly represented by the thiazolidinediones (TZDs) [52], a class of insulin-sensitizing drugs widely used in the treatment of type 2 diabetes mellitus [52, 53]. Most of the known target genes that are transcriptionally activated by PPAR*γ* belong to the pathway of metabolism and transport of lipids [42, 49]. However, PPAR*γ* may also repress gene transcription. In monocytes and macrophages, for example, it reduces IL-4 (interleukin-4) expression. Also, it reduces proinflammatory proteins, like (tumor necrosis factor *α*) TNF-*α*, (interleukin-1) IL-1, (inducible nitric oxide synthetase) iNOS, and proinflammatory transcription factors such as (activator protein-1) AP-1, (signal transducer and activator of transcription) STAT, and (nuclear factor-kB) NF-kB [54–56]. The mechanisms by which this occurs is unclear. It is generally accepted that in the unliganded state, PPAR*γ* can form weak interactions with (nuclear receptor corepressor) NcOR and (silencing mediator of retinoid and thyroid hormone receptors) SMRT to repress target gene expression [57]. Target genes that mediate the anticancer activity of the activated PPAR*γ* are still largely undefined and can be related to a wide range of processes including tumour cell differentiation, apoptosis, anti-proliferative effects, and modulation of angiogenic phenotype of the tumour microenvironment.

## 5. Effects of PPAR*γ* on the IR Gene

Studies aimed at understanding whether IR could constitute a target gene for PPAR*γ* have been performed in several cultured cell lines expressing variable amounts of PPAR*γ*. In HepG2 human hepatoma cells IRs are relatively abundant, while endogenous PPAR*γ* is barely detectable. Protein expression studies showed that IR protein content was considerably reduced in HepG2 cells overexpressing PPAR*γ* (~50% less than control cells), and this reduction was more pronounced in PPAR*γ*-overexpressing cells exposed to TZDs. It has been postulated that PPAR*γ* could affect IR content by blunting IR gene transcription. To this end, reporter gene studies have been performed using the pIR-CAT recombinant vector that contains the entire 5’-flanking region of the human IR gene [36]. Forced expression of PPAR*γ* in HepG2 cells transfected with pIR-CAT determined a ~50% reduction in CAT activity that was similar to the reduction observed in cells exposed to rosiglitazone alone, and resulted more marked in PPAR*γ*-overexpressing cells simultaneously treated with TZD rosiglitazone. These findings were confirmed in PPAR*γ* overexpressing MCF-7 human breast cancer cells, a cell line naturally expressing only detectable levels of PPAR*γ* [58], and 3T3-L1 adipocytes producing relatively high amounts of endogenous PPAR*γ* [59]. Exposure of both cell lines to either rosiglitazone and/or ciglitazone considerably reduced IR gene transcription [60]. When the recombinant plasmid pCAT-C2 was transfected into HepG2 cells (and the other cell lines), PPAR*γ*, and TZDs, either alone or in combination, inhibited pCAT-C2 activity to the same extent than they did in HepG2 cells transfected with the full-length pCAT-IR promoter, indicating that interference of PPAR*γ* and agonists with IR gene-transcription machinery occurs at the level of the proximal promoter region, C2. This conclusion was supported by the observation that in experiments using the pCAT-E3 reporter vector, containing the more distant E3 sequence of the IR gene, no effects on CAT activity were observed in cells exposed to PPAR*γ* and/or TZDs. However, no PPRE have been identified on the promoter region of the IR gene. A similar observation has been previously provided by us for the transcription factor AP-2*α*, for which DNA-binding activity was undetectable within the IR gene promoter, and transactivation of the IR gene by AP-2*α* occurred indirectly through physical and functional cooperation with HMGA1 and Sp1 [31]. Therefore, we postulated that PPAR*γ* could perturb IR gene transcription via noncanonical mechanisms interfering with the “enhanceosome” complex formation implicated in transcriptional activation of the IR gene. This hypothesis was confirmed by GST-pull-down studies, followed by electrophoresis mobility shift assay and chromatin immunoprecipitation analyses, demonstrating that PPAR*γ* physically interacted with Sp1, AP-2*α* (in selected cell lines) and, to a lesser extent, with C/EBP*β* and prevented binding of AP-2*α* to Sp1 protein, as well as of Sp1 and C/EBP*β* to their DNA consensus sites within the IR gene locus [60].

**Figure 2:**
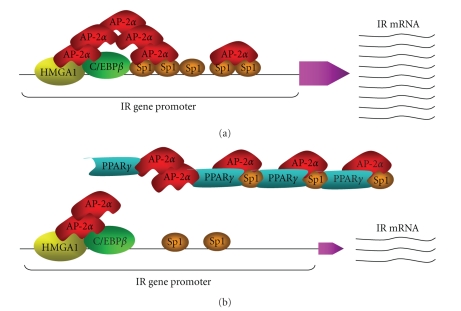
Proposed model for the action of PPAR*γ* in the context of the IR gene. AP-2*α* overexpression accounts for increased IR expression in both breast cancer cell lines and breast cancer tissues [31]. In this model, interactions between AP-2*α* and Sp1 in the preinitiation complex at the IR promoter are facilitated and stabilized by HMGA1. By binding to AP-2*α* and Sp1, PPAR*γ* may attenuate the stimulatory role of AP-2*α* in IR gene expression in breast cancer.

Thus, overexpression and/or activation of PPAR*γ* may adversely affect IR gene transcription in the absence of PPRE on the IR gene promoter. By causing a displacement of Sp1, AP-2*α*, and C/EBP*β*, PPAR*γ* may play significant molecular roles in the transcriptional activities of these factors in the context of the IR gene, both in physiology and pathology (Figure 2). These findings are consistent with the notion that IR is regulated by PPAR*γ* through PPRE-independent mechanisms. Further, they are in line with the hypothesized antineoplastic effects of PPAR*γ* and TZD, and support a potential use of PPAR*γ*-agonists as antiproliferative agents in selected neoplastic tissues, overexpressing IRs.

## 6. Discussion

The IR is critical in the insulin-mediated effects on cell metabolism and cell growth. Various studies have shown that IRs are increased in most human breast cancers, and both ligand-dependent malignant transformation and increased cell growth occur in cultured breast cells overexpressing the IR [31, 61, 62]. Also, overexpression of functional IRs has been involved in thyroid carcinogenesis [63]. In all these cases, the IR can exert its oncogenic potential in malignant cells via abnormal stimulation of multiple cellular signaling cascades, enhancing growth factor-dependent proliferation and/or by directly affecting cell metabolism.

The TZDs are insulin-sensitizing drugs that improve insulin sensitivity in insulin-resistant states such as type 2 diabetes mellitus and obesity. These drugs are high-affinity ligands for the nuclear receptor PPAR*γ*, which regulate transcription of target genes involved in the homeostasis of nutrients. In addition to the effects on lipid and glucose metabolism, many evidences have shown that PPAR*γ* and its ligands play an important role in modulating many processes, including cell proliferation and differentiation. Both breast and thyroid neoplastic cells express PPAR*γ*, and PPAR*γ* agonists have been shown to inhibit proliferation in these and other cell systems [58, 64]. In this light, studies have been performed to assess the effects of PPAR*γ* on IR expression. Results from our group have shown that IR gene transcription and expression were reduced in cells with forced PPAR*γ* expression, or TZD-induced PPAR*γ* activation. Cotreatment with both PPAR*γ* and TZDs further reduced IR protein and mRNA. In this context, treatment with specific PPAR*γ* siRNA or with PPAR*γ* antagonists should be necessary to better understand PPAR*γ*-dependent effects of TZDs. If from one hand this seems to exclude the possibility that TZDs may act as insulin sensitizers through the IR, on the other hand this results are compatible with the pleiotropic effects of PPAR*γ*. In this regard, the IR may be considered a new “target” gene that accounts for the antimitogenic response to PPAR*γ* and its agonists, and this is the first description of a cell membrane tyrosine kinase receptor involved in PPAR*γ* induced antiproliferative mechanisms. 

We showed that PPAR*γ* physically interacted with Sp1, C/EBP*β*, and AP-2*α* reducing IR gene transcription in the absence of PPAR*γ* DNA-binding sites on the IR gene. We suggested that, in the absence of PPRE in the context of the IR promoter, this nuclear receptor may produce its adverse effects on IR gene expression by interacting physically with these factors, thus reducing their availability to the basic transcription machinery of the IR gene. With a similar mechanism involving Sp1/PPAR*γ* protein interaction, PPAR*γ* has been shown to exert an antiproliferative role by suppressing transcription of the thromboxane receptor, the cyclin-dependent kinase inhibitor p21, and the fibronectin genes [65–67]. The molecular mechanism by which PPAR*γ* inhibits IR gene expression is therefore in agreement with the increasing repertoire of “noncanonical” PPAR*γ* target genes that now encompasses non-PPRE containing genes [50]. Many potential “target” genes of PPAR*γ*, regulated by DNA-independent mechanisms, have been already reported, including bcl-2 (oncogene B-cell leukemia 2, *β*-catenin, and the PTEN (phosphatase and tensin homolog) tumor suppressor gene [68–70]. However, how PPAR*γ* and its agonists may induce their antiproliferative effects is not fully understood yet. Recently, non-genomic cross-talks between PPAR*γ* and cytoplasmic proteins, like (extracellular signal regulated kinase) ERK 1/2, and (mitogen-activated protein kinase) MAPK kinases, have been reported in cancer cells and functional importance has been given to the subcellular localization of PPAR*γ* [71, 72]. In this regard, in PC-3 prostate cancer cells, IGF-1R signaling has been shown to be attenuated by rosiglitazone via non-genomic action on ERK 1/2, and protein kinase AKT phosphorylation [73]. Therefore, both nuclear and cytoplasmic events may be theoretically operative in inhibiting the mitogenic signals common to the receptors of the IGF system (IR, IGF-1R and hybrid IR/IGF-1R). 

The last three decades of medical research examining the molecular pathogenesis of cancers have provided compelling evidence for the universal disruption of the cell cycle in human tumours, and recent studies have demonstrated a critical interface between hormonal signaling and the cell cycle [74]. In this context, mitogens like insulin, via the IR, may promote the progression through the G1 phase by inducing competence of the cyclin D1/cyclin-dependent kinase 4 (CDK4) complex. It has been previously reported that the PPAR*γ* agonists inhibit cyclin D1 [75]. Our findings on IR and PPAR*γ* support the conclusion that PPAR*γ* and TZDs may interfere with the hormonal control of the cell cycle, at least in part, through the inhibition of the IR. Over the last decade, PPAR*γ* has emerged as an important drug target in type 2 diabetes mellitus [76], and TZDs are widely used for treatment of diabetic patients. However, conflicting results on the procarcinogenic and antitumorigenic effects of TZDs in humans with diabetes can be found in the literature. For instance, whereas a population based report showed that TZDs were associated with reduced risk of lung cancer in patients with diabetes [77], a possible association between cancer and the use of TZD has been reported later in type 2 diabetic patients [78]. Thus it is tempting to suppose that IR gene may be considered a new anticancer target for PPAR*γ*, providing further evidence for the use of TZDs as anti-proliferative agents in selected tumours overexpressing the IR.
